# Editorial: The impact of sex and gender on disease diagnostics in global health

**DOI:** 10.3389/fgwh.2026.1969320

**Published:** 2026-10-09

**Authors:** Elena Marbán-Castro, Xiaohui Sem, Elena Dacal, Maria Cristina Manzanares-Céspedes

**Affiliations:** 1 Global Health Research Group, University of the Balearic Islands (UIB), Palma, Spain; 2 Women in Global Health Spain, Barcelona, Spain; 3 Woodseer Resources, Kuala Lumpur, Malaysia; 4 Women in Global Health Singapore, Singapore, Singapore; 5 Spotlab, Madrid, Spain; 6 University of Barcelona, Barcelona, Spain

**Keywords:** diagnosis, gender identity, global health, implementation science, infectious diseases, intersectional framework, non-communicable diseases, sex characteristics

Sex and gender shape health in ways that medicine has been slow to measure. Sex, the biological attributes that differ between female, male, and intersex individuals, and gender, the socially constructed roles, norms, and relations that vary across societies, as women, men, and others, both influence who becomes ill, how illness manifests, who seeks care, who is tested, and who is diagnosed and treated. Gender is recognized as one of the central social determinants of health, yet diagnostic pathways, the point at which a symptom becomes a condition, remain among the least examined stages of the care continuum. Interestingly, “gender” as a concept does not exist as a MeSH term. The term “gender” always appears in association with other concepts, such as “gender-affirming care” or “gender identity.” This absence is telling: even in terms of how scientific knowledge is organized, gender is treated as a modifier rather than a determinant in its own right.

We launched this Research Topic to bring that stage into focus. We aimed to gather evidence on how sex, gender, and their intersections with other social determinants of health, such as age, socioeconomic status, ethnicity, disability, and geography, affect the diagnosis of infectious and non-communicable diseases (NCDs). The 12 articles collected here span basic immunology, clinical epidemiology, global burden modeling, health services research, and community-based fieldwork. Together, they make a consistent case: ignoring sex and gender in diagnosis is not a neutral omission but an active driver that reproduces health inequalities in society.

Several contributions trace the biological roots of sex differences that diagnostics too often overlook. Lodde et al. examined how X chromosome-encoded microRNAs regulate immune responses, offering a mechanistic explanation for the sex disparities seen across infectious and autoimmune diseases. Hong et al. reviewed the role of estrogen in bacterial infection, showing how the hormonal context can alter susceptibility and, by extension, the interpretation of diagnostic findings. These reviews are a reminder that reference ranges and risk models built on male-default physiology may contribute to diagnostic inaccuracies in women.

Cardiovascular disease, long framed as a male problem, emerges as a recurring theme. Dakhil et al. argued for rethinking cardiovascular risk prediction in women through precision medicine, challenging current diagnostic tools that systematically underestimate the risk faced by women. López-Ferreruela et al. showed, in a large Spanish cohort, that women receive less intensive monitoring and follow-up after acute myocardial infarction, a gap that begins with under-recognition and propagates throughout the care pathway. Gao et al. added a preventive dimension, analyzing sex-specific associations between dietary carotenoid intake and heart failure risk using the United States’ National Health and Nutrition Examination Survey data. Together, these studies show that closing the cardiovascular gender gap requires attention at every stage, from risk estimation to secondary prevention.

The global burden of chronic conditions is examined through several large-scale analyses. Li et al. modeled the global burden of polycystic ovary syndrome from 1990 to 2021 with projections to 2035, quantifying a condition whose diagnosis is frequently delayed. Liu et al. mapped gender disparities in occupational noise-induced hearing loss, a reminder that occupational exposures and the diagnostics that capture them are themselves gendered. Faulstich et al. identified sex differences in clinical risk factors for early- and late-onset Alzheimer's dementia, informing more tailored diagnostic assessment. These studies share a methodological strength: they disaggregate by sex rather than adjusting it away.

Therapeutics and surgical care are not exempt. Hu et al. conducted a scoping review of sex- and gender-specific differences in the efficacy and safety of traditional Chinese medicine, an area in which such analyses remain rare. Ripoll-Romero et al. reported gender differences among patients presenting for hip arthroplasty, showing how clinical presentation and referral can diverge well before the operating room.

Two contributions speak directly to the infrastructure and equity of diagnosis in global health settings. Atiomo et al. described a study protocol to establish a dedicated women's health biobank in Dubai, as sex- and gender-stratified diagnostics depend on representative resources. Gandigbe et al. took an intersectional approach, examining how gender and disability interact to shape inequities in neglected tropical disease-endemic communities in Benin and Côte d’Ivoire. Their study grounds the Research Topic within settings where diagnostic gaps carry the highest cost.

A few threads run across these diverse articles. The first is that sex and gender operate together, not in isolation: biology sets differential susceptibility, while gender norms govern whether a person reaches a clinic, is believed, and is tested. The second is intersectionality. As Gandigbe et al. make clear, gender rarely acts alone; disability, poverty, and geography compound one another, further proving gender as one of the main socioeconomic causes of health status disparities. The third is the persistent problem of the male default, in reference ranges, risk scores, biobanks, and clinical suspicion, which quietly shapes who is diagnosed, how, and when.

We are honest about what this collection does not yet resolve. Much of the evidence remains observational, and several studies analyze sex and gender as binary variables, leaving the diagnostic experiences of intersex, transgender, and gender-diverse people largely unaddressed. Data from low- and middle-income countries, where diagnostic capacity is scarcest, remain under-represented. Furthermore, while many studies document disparities, test interventions and implementation processes to close these gaps lie remain areas that future work must address.

Still, the direction is clear. Better diagnostics will not come from adding sex and gender as an afterthought but by designing studies, tools, and health systems that expect differences from the start. This requires sex- and gender-disaggregated reporting to become standard practice, along with gender-sensitive care pathways, representative biobanks, and diagnostic technologies that are validated across the populations that will use them. We hope that this Research Topic will offer evidence and momentum for this work, and we thank the authors, reviewers, and the Frontiers editorial team for making it possible.

